# Treatment of vulvar cancer recurrence with electrochemotherapy: a case-control study

**DOI:** 10.2340/1651-226X.2024.33241

**Published:** 2024-05-21

**Authors:** Gregor Vivod, Masa Omerzel, Nina Kovacevic, Gorana Gasljevic, Ines Cilensek, Gregor Sersa, Maja Cemazar, Sebastjan Merlo

**Affiliations:** a Department of Gynecological Oncology, Institute of Oncology Ljubljana, Ljubljana, Slovenia; b Faculty of Medicine, University of Ljubljana, Ljubljana, Slovenia; c Department of Experimental Oncology, Institute of Oncology Ljubljana, Ljubljana, Slovenia; d Faculty of Pharmacy, University of Ljubljana, Ljubljana, Slovenia; e Department of Pathology, Institute of Oncology Ljubljana, Ljubljana, Slovenia; f Faculty of Medicine, University of Maribor, Maribor, Slovenia; g Institute of Histology and Embryology, Faculty of Medicine, University of Ljubljana, Ljubljana, Slovenia; h University of Ljubljana, Faculty of Health Sciences, Ljubljana, Slovenia; i Faculty of Health Sciences, University of Primorska, Izola, Slovenia

**Keywords:** Vulvar cancer, recurrence, effectiveness, electrochemotherapy, bleomycin, wide excision

## Abstract

**Background:**

Electrochemotherapy (ECT) is a combined treatment method based on electroporation and simultaneous chemotherapy. In cases where radiotherapy has previously been used, surgery is often the only treatment option for vulvar cancer recurrence with potential resection of clitoris, vagina, urethra or anal sphincter. The unique advantage of ECT is its selectivity for cancer cells while sparing the surrounding healthy tissue. The aim of the study was to compare the ECT treatment of vulvar cancer recurrence for non-palliative purposes with surgical treatment.

**Materials and methods:**

Eleven patients with single vulvar cancer recurrence were treated with ECT and followed up for 12 months. As a control group, 15 patients with single vulvar cancer recurrence were treated with wide local excision. The following data were collected, analyzed and compared: Age, body mass index, comorbidities, histological type, location and size of vulvar cancer recurrence, treatment history, details of procedures and hospital stay.

**Results:**

The probability curves for local tumor control did not differ between the ECT group and the surgical group (*p* = 0.694). The mean hospital stay and the mean duration of procedure were statistically significantly shorter in the ECT group (*p* < 0.001). There were no statistically significant differences between the ECT and surgical groups in terms of mean body mass index, associated diseases, previous treatments, presence of lichen sclerosus, p16 status, gradus, anatomical site of the tumor, and type of anesthesia.

**Conclusion:**

In this case-control study, treatment of vulvar cancer recurrence with ECT for non-palliative purposes was comparable to surgical treatment in terms of effectiveness. The results need to be confirmed in larger randomized trials.

## Introduction

Vulvar cancer is a rare disease and accounts for about 5% of all gynecologic cancers [1]. In recent years, there has been a statistically significant increase in the incidence of vulvar cancer in young women worldwide, which can be attributed to HPV infection [1, 2]. Squamous cell carcinomas account for more than 90% of vulvar cancer cases [3]. Surgery and radiotherapy are currently the main treatment modalities for vulvar cancer. Treatment decisions for vulvar cancer depend on staging, performance status, patient age, and the presence of comorbidities [4]. The reported overall recurrence rate of vulvar cancer is 37%, the majority of which are local recurrences [5]. The treatment of local recurrences is usually challenging and requires an individualized approach, depending on the site of recurrence and previous treatment. In recent years, the approach to treating vulvar cancer has evolved from invasive surgery to more conservative approaches, that are as individualized as possible, with the integration of new surgical techniques [6].

Electrochemotherapy (ECT) is a combined modality treatment based on electroporation and concurrent chemotherapy, usually bleomycin, administered intravenously [7]. It is already used for the treatment of superficial tumors such as melanoma, sarcoma, squamous cell carcinoma, basal cell carcinoma, and also for the treatment of deep-seated tumors, such as primary hepatocellular carcinoma and colorectal liver metastases [8–12]. For deep-seated tumors, not only open surgery approach, but also percutaneous and laparoscopic methods have been developed [13, 14]. The unique advantage of ECT is its selectivity for cancer cells while sparing surrounding healthy tissue [15]. To date, the use of ECT in patients with vulvar cancer has been described only in a few studies or clinical cases and only for palliative purposes [16–19].

In 2023, our group published for the first time the data of a study in which ECT was used to treat the recurrence of vulvar cancer for non-palliative purposes. ECT has been shown to be a feasible and safe technique for the treatment of vulvar cancer recurrence [20]. The aim of the study was to compare the ECT treatment of vulvar cancer recurrence for non-palliative purposes with surgical treatment.

## Methods

### Study design

The study on the treatment of vulvar cancer recurrence for non-palliative purposes with ECT was a case-control study, prospective and institution-based. The data from patients in whom vulvar cancer recurrence was treated with surgery were collected retrospectively. The study was conducted at the Institute of Oncology in Ljubljana and was approved by the Institutional Medical Board (number ERID–KSOPKR–0042/2021) and the Slovenian National Ethics Committee (number 0120–262/2021/3). The study was registered at ClinicalTrials.gov under the identification number NCT05916690. All patients were presented to an interinstitutional tumor board consisting of a gynecologic oncologist, a medical oncologist, a radiation oncologist, a radiologist, and a pathologist. A signed informed consent form was obtained from all patients included in the study.

### Patients and data collection

From July 2020 to January 2023, 11 patients with vulvar cancer recurrence were treated with ECT. Patients were included in the study according to the inclusion and exclusion criteria [20]. A cohort of 15 patients with vulvar cancer recurrence who were treated with wide local excision from January 2018 to January 2023 was selected as a control group. All patients in both groups had a single local vulvar cancer recurrence confirmed by an experienced pathologist. Regional and distant metastases were excluded by appropriate imaging. The data collected were anonymized to protect the privacy of all patients. Specifically, the following data were analyzed and collected: Age, body mass index, comorbidities, histological type, location and size of recurrence, treatment history, treatment data including details of procedures and hospital stay. When possible, a photographic image of the lesions before and after ECT was obtained.

### Treatment procedures, response evaluation and follow up

ECT was performed according to standard operating procedures [21]. Bleomycin was administered intravenously at a dose of 10,000 or 15,000 IU/m^2^ (Bleomycin Medac, Medac GmbH,Wedel, Germany), depending on the patient’s age and concomitant diseases [22]. Eight minutes after the intravenous administration of the drug, electrical impulses were applied to the tumors via specially designed electrodes in such a way that the entire tumor node, including a safety margin of one centimeter, was covered. The electrical impulses were generated using the Cliniporator VITAE (IGEA S.p.A., Carpi, Italy). In a control group, the wide excision of tumor was made with macroscopic safety margin of one centimeter.

Effectiveness of ECT was evaluated by clinical response based on the Response Evaluation Criteria in Solid Tumors (RECIST) version 1.1 criteria at 1, 2, 3, 6, 9, and 12 months after the procedure [23]. Disappearance of the lesion was defined as a complete response (CR), a decrease of at least 30% of the maximum diameter of the lesion was defined as partial response (PR) and an increase of at least 20% of the maximum diameter of the lesion was defined as progressive disease (PD). Stable disease (SD) was defined between the criteria PR and PD. In case of incomplete clinical response at 1 or 2 months after ECT, a biopsy of the lesion was performed to differentiate between tumor necrosis and residual disease. In case of residual disease 2 months after ECT, the patient was presented to an interinstitutional tumor board to consider other treatment options. Patients treated with wide local excision were clinically examined 1, 3, 6, and 12 months after surgery. In case of suspicious lesions, biopsy was performed.

### Statistical analyses

Statistical analysis was performed using IBM SPSS Statistics 27.0 software (Armonk, NY: IBM Corp). Continuous clinical data were compared using the unpaired Student’s *t*-test, while the chi-square (χ^2^) test was used to compare categorical variables. Given the potential for small sample sizes in some categories, Fisher’s exact test was used to assess associations between categorical variables such as associated diseases, types of anesthesia, and p16 status. Data were presented as mean ± SD (continuous variables) or as number and percentage of patients (categorical variables). A *p* < 0.05 was considered statistically significant. The Shapiro–Wilk test was performed to assess the normality of the distribution for all continuous variables due to the small sample dataset. This test was chosen due to its appropriateness for small sample sizes and its sensitivity in detecting deviations from normality. After confirming that the data were normally distributed (*p* > 0.05), Student’s *t*-test was used to compare means between groups. Local tumor control was estimated as a function of time using the Kaplan–Meier product limit method. Local vulvar cancer recurrence represented the end point of interest and was therefore scored as an event. Data from patients who had a complete response to therapy at the end of the follow-up period, died for other reasons, or had regional or distant metastases were used as censored data. The log-rank test was used to analyze the difference between the curves.

## Results

### Patients and tumor characteristics

Eleven patients with local vulvar cancer recurrence were treated with ECT (cases) and 15 with the surgical procedure of wide local excision (control). The patients treated with ECT were older, with a mean age of 78.4 (SD ± 9.0) years, than the surgically treated group with a mean age of 67.8 (SD ± 8.9) years. The difference between the mean age of the cases and the controls was statistically significant (*p* = 0.006). The mean body mass index of the patients was 26.1 (SD ± 3.9) in the ECT group and 26.8 (SD ± 5.9) in the surgical group. All patients in the surgical group had associated diseases, while two patients in the ECT group had no associated diseases. Five patients were treated for vulvar cancer with surgery alone before ECT, while six patients were treated with surgery and radiotherapy before ECT. In the surgical group, nine patients were treated primarly with surgery, while six patients were treated with surgery and radiation therapy. All patients in both groups had a single lesion of squamous cell carcinoma. There were five patients with lichen sclerosus in the ECT group and 11 in the surgical group. Imunohistochemistry for p16 was positive in three patients in the ECT group and in three patients in the surgical group. There were no statistically significant differences between the ECT and surgical groups in mean body mass index, associated diseases, previous treatments, presence of lichen sclerosus, and p16 status. The detailed data of the patients and the tumor characteristics are shown in Table 1.

**Table 1:** Characteristics of the patients treated with electrochemotherapy and with surgery.

Characteristics	Electrochemotherapy (*n* = 11)			Surgery (*n* = 15)			*P*
	Mean ± SD	*n*	%	Mean ± SD	*n*	%	
Age at treatment (mean ± SD)	78.4 ± 9.0			67.8 ± 8.9			0.006
BMI (mean ± SD)	26.1 ± 3.9			26.8 ± 5.9			0.758
Duration of procedure (minutes, mean ± SD)	17.6 ± 3.0			56.1 ± 20.5			< 0.001
Days of hospital stay (mean ± SD)	2.7 ± 1.1			7.5 ± 3.7			< 0.001
Longest diameter of tumor (mm, mean ± SD)	19.5 ± 13.9			26.7 ± 13.3			0.192
Number of patients with associated diseases, *N* (%)		9	81.8		15	100	0.169
Previous treatment							
Surgery		6	54.5		9	60.0	0.548
Surgery and Radiotherapy		5	45.5		6	40.0	
Number of patients with lichen sclerosus, *N* (%)		5	45.5		11	73.3	0.228
Gradus, *N* (%)							
1		1	9.1		5	33.3	0.305
2		8	72.7		7	46.7	
3		2	18.2		3	20.0	
Anatomical site of tumor, *N* (%)							
Clitoris		1	9.1		2	13.3	0.506
Paraurethral		3	27.3		3	20.0	
Labia minora		3	27.3		4	26.7	
Labia majora		2	18.2		5	33.3	
Perineum		2	18.2		1	8.3	
Anesthesia, *N* (%)							
Local		4	36.4		8	53.3	0.453
General		7	63.6		7	46.7	
p16 status, *N* (%)							
Positive		3	27.3		3	20.0	0.509
Negative		8	72.7		12	80.0	

### Tumor response to ECT

**Figure 1:**
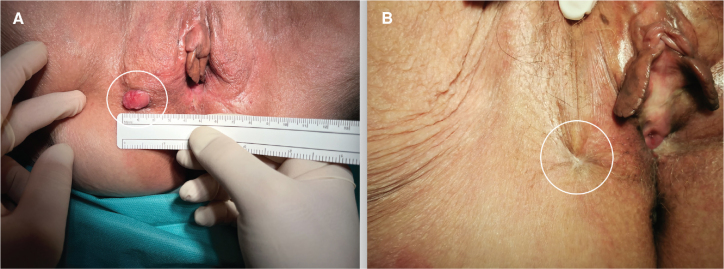
(A) Local recurrence of vulvar cancer (circled line). (B) Complete response 12 months after treatment with electrochemotherapy (circled line).

Tumor response was assessed 1, 2, 3, 6, 9 and 12 months after ECT. One month after ECT, necrosis of the tumor was evident in nine patientsand destruction of the tumor lesion in these patients. A biopsy was taken from all nine patients, which confirmed necrosis without cancer cells. One patient had a complete clinical response and one patient had stable disease. Two months after ECT, there were seven complete clinical responses with disappearance of the tumor, three lesions with necrosis from which biopsies were taken and confirmed necrosis without cancer cells. In one patient, who still had stable disease after 2 months, confirmed by biopsy with residual cancer cells, a wide local excision with free surgical margins was performed. Three months after ECT, 10 patients had a complete clinical response and no evidence of disease. Six months after ECT, there were seven patients with complete clinical response, two patients with recurrence of cancer in the vulvar region and one patient with disease progression in the pelvis with no signs of disease at the ECT treatment site. Nine months after ECT, six patients had a complete clinical response, and one patient died from other causes. Twelve months after ECT, there were six patients with complete clinical response after ECT treatment, with no signs of disease. The case of vulvar cancer recurrence treated with ECT and complete clinical response to treatment after 12 months is shown in Figure 1.

### Control group of patients treated with surgery

Patients treated with wide local excision were examined 1, 3, 6, and 12 months after surgery. One and three months after surgery, there were no signs of disease in any of the patients. One month after surgery, wound dehiscence was observed in eight patients. The wound dehiscence healed by secondary intention. Six months after surgery, there were four local recurrences of vulvar cancer, and 12 months after surgery, there was a local recurrence of vulvar cancer in three more patients.

### Comparision of groups with treatment details

**Figure 2:**
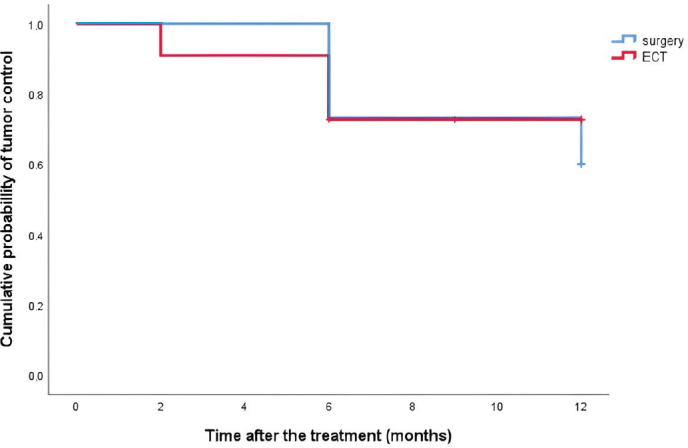
Kaplan–Meier curves for the local tumor control probability for patients treated with surgery or electrochemotherapy (ECT).

Mean diameter of the tumor in the ECT group was 19.5 (SD ± 13.9) millimeters and 26.7 (SD ± 13.3) millimeters in the surgical group, which was not statistically significant for the type of treatment (independent sample *t*-test, *p* = 0.192). Mean hospital stay was 2.7 (SD ± 1.1) days in the ECT group and 7.5 (SD ± 3.7) days in the surgical group. Mean duration of procedure was 17.6 (SD ± 3.0) minutes in the ECT group and 56.1 (SD ± 20.5) minutes in the surgical group. Mean hospital stay and mean duration of procedure were statistically significantly shorter in the ECT group (independent sample *t*-test, *p* < 0.001 for both groups). Four patients were treated under local anesthesia in the ECT group and eight patients in the surgical group, while seven patients were treated under general anesthesia in the ECT group and surgical group. There were no statistical differences between the ECT and surgical groups in gradus, anatomical site of the tumor, and anesthesia. The detailed data of the treatment details are shown in Table 1. The probability curves for local tumor control did not differ between the ECT group and surgical group (Figure 2; Longrank test, *p* = 0.694).

## Discussion

In this case-control study, treatment of vulvar cancer recurrence with ECT for non-palliative purposes provided similar local control to surgical treatment. The effect of treatment with ECT was evaluated by clinical response based on the RECIST version 1.1 criteria, which are based on one-dimensional clinical measurements [23]. The criteria have proven to be a suitable tool for assessing the response to ECT. The surgically treated patients were clinically assessed at regular follow-up visits according to the guidelines for vulvar cancer [24]. The difference in the assessment of clinical response is due to the immediate removal of the tumor during surgery and the delayed destruction of cancer cells after treatment with ECT, which usually takes up to 6 weeks [21]. At 12 months after treatment, there were 6 patients (54.4%) with complete response to ECT and 8 patients (53.3%) with no evidence of disease after surgical treatment of vulvar cancer recurrence. In the study with the longest follow-up in which ECT was used for palliative purposes in vulvar cancer, 32% of patients with a complete response were disease-free after 2 years [19]. In other studies in which ECT was used for palliative purposes in vulvar cancer, complete response rates ranged from 45.2 to 53.3%, with a median follow-up of 6–12 months [16–18]. In the largest study of squamous cell carcinomas treated with ECT in general (non-vulvar carcinomas), a complete response was achieved in 63% of cases, but with a short average follow-up of at least 45 days [8]. Population-based studies of recurrent vulvar cancer treated surgically for non-palliative purposes are sparse and results from a nationwide cohort are lacking. However, the median 2-year survival rate after isolated vulvar cancer recurrence is 57.8% [5].

The patients treated with ECT in the study were older, which shows that ECT is well tolerated and effective in the elderly population with associated diseases. The anatomical site of vulvar cancer recurence was equally distributed in both groups, and in both the clitoris, vagina, urethra and anus were affected in close proximity. The nuclear grade of all squamous cell carcinomas and the p16 status were equally distributed and did not influence the efficacy of the treatment in both groups. The advantages of treating vulvar cancer recurrence with ECT compared to surgical treatment were the shorter duration of the procedure and the shorter hospital stay. Based on the safety results of a previous studies of treatment of vulvar cancer with ECT, the patient could be admitted and discharged from the hospital on the same day [16, 20]. The shorter duration of ECT is the only need to arrange the electrodes appropriately to deliver the electrical pulses throughout the tumor area with the surgical safety margin [21]. Once the electrodes are removed, there is no or minimal bleeding and the treatment is complete. Surgical treatment requires adequate wide local excision, which may be associated with major bleeding and adequate suture reconstruction, which prolongs the surgery time [24, 25].

Another advantage of treating vulvar cancer recurrence with ECT is the avoidance of mutilating surgery. In cases where radiation has been previously performed, surgery is often the only treatment option for vulvar cancer recurrence with potential resection of the clitoris, part of the vagina, urethra or anal sphincter. The surgeon had to compromise on the surgical safety margin or make the decision to perform a pelvic exenteration, which is associated with high morbidity and mortality [26]. In ECT, the entire area of the tumor and the surgical safety margin of one centimeter are covered with an appropriate selection of electrodes [20, 21]. This is particularly important in the recurrence of vulvar cancer near the clitoris, vagina, anus, or urethra, where ECT destroys the cancer cells but spares the tissues and organs around the tumor. This has already been demonstrated in local treatment with ECT in patients with head and neck tumors, where radiotherapy and surgery can be very strenuous for the patient and may result in functional limitations that severely impair the patient’s quality of life. In up to 96% of cases, the examined region on the head and neck almost resembled the normal surrounding skin after treatment with ECT and showed excellent results in terms of restoring the original condition without deformation or distortion [15, 27]. In some patients, the healing phase can be prolonged to several weeks, depending on the healing potential [28]. Therefore, if vulvar cancer recurs near the clitoris, vagina, urethra, or anus, and the one centimeter safety margin area is crucial, ECT may be the better treatment choice as it preserves healthy tissue, while surgical excision could result in impaired urination, defecation or sexual intercourse.

After ECT, tissue healing occurs by secondary intention over 4–8 weeks, depending on tumor size and response [29]. There are no restrictions on showering after treatment, which is very important for vulvar area [21]. Pain is treated with mild analgesics or opioids depending on the severity [7]. Similarly, wound dehiscence or infection in the vulvar area occurs in 20–58% of cases after surgical treatment of vulvar cancer, which also requires a specialized care team with appropriate pain management [30]. The complication rate for pelvic exenteration is between 57 and 82% of cases. The most common complications are bleeding, ileus, wound complications, respiratory failure and acute renal failure [26, 31–33].

Women who undergo repeated surgical treatment for vulvar cancer may experience narrowing of the introitus or vagina due to scarring, leading to sexual dysfunction and dissatisfaction with the partner relationship [34]. The extreme changes in the anatomy of the vulva can lead to dyspareunia, negative feelings about body image and the inability to achieve orgasm [35, 36]. Surgical treatment in vulvar area has such a profound effect on sexual function that many women abandon intercourse completely after a vulvectomy. These women are more likely to experience depressive and anxiety symptoms, and their lives are negatively affected by shame and insecurity [35–37]. In the case of pelvic exenteration, the creation of a bowel or urinary stoma can have an enormous impact on the patient’s quality of life and seriously affect social relationships. These inconveniences are particularly severe in younger patients [34]. In this sense, ECT may lead to an improved quality of life for patients, as the surrounding healthy tissue is spared compared to surgery and the anatomy of the vulva is less altered.

The study has several strengths. Firstly, this study is the first to provide information about effectiveness of ECT in vulvar cancer recurrence for non-palliative purposes. Secondly, the patients included in the ECT group and the surgical group were patients with similar clinical characteristics and were homogeneous in terms of the same histological type, previous treatments, and anatomical sites. Thirdly, our center is an important referral center for ECT treatment and has important experience [9, 12, 14, 38, 39].

The main limitation of the study is the small number of patients treated. However, vulvar cancer recurrences are rare and difficult to treat. The results of the study, including those derived from the significance tests, should be considered preliminary. They serve as a basis for generating hypotheses and as a guide for future studies with larger samples, where the risk of random significance can be further mitigated. Therefore, small case-control studies are also important to test the value of new treatment methods. The second limitation was the relatively short follow-up time, but it was long enough to prove the efficacy of the treatment, which was the main objective of the study. The third limitation of the study is the retrospective data collection in the surgical group. The risk of bias was minimized by including all patients who had a single lesion with vulvar cancer recurrence and the same inclusion and exclusion criteria as the ECT group and were treated with wide excision between January 2018 and January 2023.

In summary, in the study there was no statistically significant difference in local tumor control between the group treated with surgery and the group treated with ECT. The study shows that treatment with ECT of vulvar cancer recurrence for non-palliative purposes is comparable to surgical treatment in terms of effectiveness and might be used in cases involving the proximity of the clitoris, vagina, urethra and anus.

## Conclusion

ECT is easy and quick to perform and is associated with a short hospital stay. ECT appears to be less mutilating and may be a promising treatment alternative for vulvar cancer recurrence, especially in cases where the clitoris, vagina, urethra and anus are in close proximity. As this is the first study in which vulvar cancer recurrence was treated with ECT for non-palliative purposes, there is no data on patients’ quality of life compared to surgery. Detailed studies on the quality of life of patients with vulvar cancer recurrence are lacking and are therefore recommended. In addition, further analyses with a longer observation period are required to evaluate the effectiveness of ECT in vulvar cancer recurrence compared to surgery.

## Data Availability

Corresponding author can be contacted for access to data and will provide data upon reasonable request.
